# Correction: Marigold-flower-like MoS_2_ nanosheet assemblies for enhanced alkaline hydrogen evolution

**DOI:** 10.1039/d6ra90035f

**Published:** 2026-04-01

**Authors:** Rajvardhan K. Chougale, Prashant D. Sanadi, Sanket N. Yadav, Srinivaas Masimukku, Guo-Ping Chang-Chien, Babasaheb D. Bhosale, Ganesh S. Kamble

**Affiliations:** a Department of Engineering Chemistry, Kolhapur Institute of Technology's College of Engineering (Empowered Autonomous), Kolhapur Affiliated to Shivaji University Kolhapur 416234 India ganeshchemistry2010@gmail.com kamble.ganesh@kitcoek.in; b School of Nanoscience and Technology, Shivaji University Kolhapur 416004 India; c Center for Environment Toxin and Emerging-Contaminant Research, Cheng Shiu University Kaohsiung Taiwan 833301 Republic of China; d Institute of Environment Toxin and Emerging-Contaminant, Cheng Shiu University Kaohsiung Taiwan 833301 Republic of China; e Conservation and Research Center, Cheng Shiu University Kaohsiung Taiwan 833301 Republic of China; f Department of Chemistry, Rajaram College Kolhapur India bdb.bhosale@gov.in

## Abstract

Correction for ‘Marigold-flower-like MoS_2_ nanosheet assemblies for enhanced alkaline hydrogen evolution’ by Rajvardhan K. Chougale *et al.*, *RSC Adv.*, 2026, **16**, 14547–14554, https://doi.org/10.1039/D6RA00011H.

The authors regret that the original article contained an error in the Electrochemical measurements section on page 14549, whereby ‘ZCF-*x*’ should be replaced with ‘MoS_2_’.

The text originally read “Nickel foam (1 × 1 cm^2^) served as the working electrode and was coated with catalyst ink prepared by ultrasonically dispersing 20 mg of ZCF-*x* in a mixed solvent containing 796 µL of deionized water, 200 µL of isopropyl alcohol, and 4 µL of Nafion solution.”.

This sentence has been corrected to “Nickel foam (1 × 1 cm^2^) served as the working electrode and was coated with catalyst ink prepared by ultrasonically dispersing 20 mg of MoS_2_ in a mixed solvent containing 796 µL of deionized water, 200 µL of isopropyl alcohol, and 4 µL of Nafion solution.”.

The Royal Society of Chemistry apologises for these errors and any consequent inconvenience to authors and readers.

